# Diagnosis and treatment of gastric hamartomatous inverted polyp (GHIP) using a modified combination of laparoscopic and endoscopic approaches to neoplasia with a non-exposure technique (modified CLEAN-NET): a case report

**DOI:** 10.1186/s40792-020-00951-5

**Published:** 2020-08-06

**Authors:** Suguru Hayase, Mei Sakuma, Shun Chida, Masaru Saito, Hirofumi Ami, Yoshihisa Koyama, Shinji Ohki, Koji Kono

**Affiliations:** 1 Department of Surgery, Ohara General Hospital, 6-1, Uwa-machi Fukushima-shi, Fukushima, 960-8101 Japan; 2 Department of Gastrointestinal Tract Surgery, Fukushima Medical University, 1, Hikarigaoka Fukushima-shi, Fukushima, 960-1295 Japan

**Keywords:** Gastric hamartomatous inverted polyp, Modified CLEAN-NET, Non-exposure technique, LECS, Submucosal tumor

## Abstract

**Background:**

Gastric hamartomatous inverted polyp (GHIP) is a pathological condition where enlarged gastric glands with cystic dilatation grow in the submucosa. It is difficult to excise the tissue due to its location. In addition, even if the tissue is taken correctly, making an accurate diagnosis is difficult due to foveolar epithelium in the tissue, which can be misdiagnosed as gastric mucosal epithelium. Thus, an accurate diagnosis of GHIP is rarely established from a biopsy alone preoperatively. We here report a case of GHIP with a central dimple, which was diagnosed and treated using a modified combination of laparoscopic and endoscopic approaches to neoplasia with a non-exposure technique (modified CLEAN-NET).

**Case presentation:**

A 60-year-old man with a submucosal tumor (SMT) in the stomach was referred to our hospital by a primary care doctor. On examination, a gastrointestinal stromal tumor was suspected. Modified CLEAN-NET was performed for diagnostic and therapeutic purposes. The histopathological examination of the resected specimen showed an enlarged gland duct in the submucosal layer. This finding, along with immunostaining results, led to the diagnosis of GHIP. The postoperative course was uneventful without any symptoms.

**Conclusions:**

GHIP should be considered among the differential diagnoses of SMT of the stomach. Modified CLEAN-NET may be beneficial in the removal of SMTs such as GHIP with a central dimple because it can avoid stomach deformation of the stomach and tumor dissemination.

## Background

Gastric hamartomatous inverted polyp (GHIP) is characterized by marked submucosal glandular proliferation associated with cystic dilation [1]. It is difficult to diagnose preoperatively because the main lesion is located in the submucosa or within the muscularis mucosae [2]. As such, diagnostic resections have been performed in many cases of GHIP. Such resections are performed by endoscopic submucosal dissection (ESD) or laparoscopic and endoscopic cooperative surgery (LECS) in clinical practice, because GHIP is considered a benign disease [3–5]. However, although relatively uncommon, it has been reported GHIP occasionally contains gastric cancer [5, 6] and can manifest as a central dimple or umbilication [7]. Therefore, the tumor has to be prevented from exposure to the abdominal cavity in order to prevent dissemination when LECS is conducted. To avoid intraoperative tumoral dissemination, a combination of laparoscopic and endoscopic approaches to neoplasia with a non-exposure technique (CLEAN-NET) was developed [8]. Here, we present a case of GHIP with a central dimple, which was diagnosed and treated using modified CLEAN-NET.

## Case presentation

**Fig. 1:**
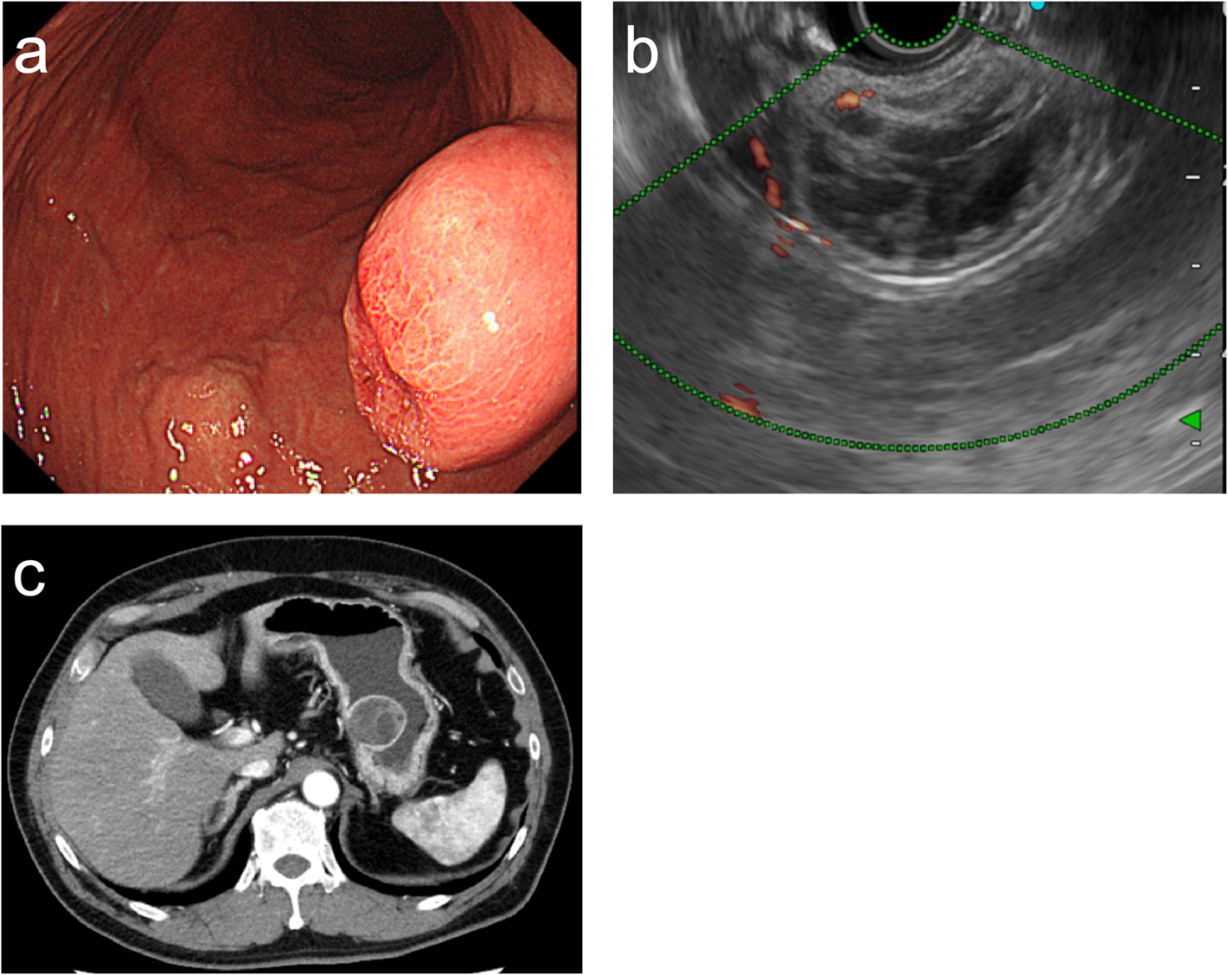
**a** Upper gastrointestinal endoscopy showed a gastric SMT with a central dimple at the posterior wall of the upper gastric body. **b** Endoscopic ultrasonography (20 MHz) showed heterogenous tumor measuring 35 mm in diameter with cystic spots. **c** An enhanced computed tomography scan revealed an intraluminal growth type tumor at the posterior wall of the upper gastric body

An asymptomatic 60-year-old man was referred to our hospital because of a submucosal tumor (SMT) of his stomach found by an upper gastrointestinal endoscopy at a regular health check. The patient had been on medications for hypertension and old cerebral infarction. The upper gastrointestinal endoscopic examination revealed a gastric SMT with a central dimple at the posterior wall of the upper gastric body (Fig. 1a). Endoscopic ultrasonography (20 MHz) showed a heterogenous tumor measuring 35 mm in diameter with cystic spots. However, it was unclear on which ultrasonography layer the lesion of the tumor was located (Fig. 1b). An enhanced computed tomography scan revealed an intraluminal growth type tumor at the posterior wall of the upper gastric body. There was no evidence of perigastric invasion, lymphadenopathy, or distant metastasis (Fig. 1c). Based on the imaging results, a gastrointestinal stromal tumor (GIST) was suspected, and the patient was referred to our hospital for diagnostic resection of the SMT. Modified CLEAN-NET for gastric local resection was selected in order to avoid stomach deformation and tumor dissemination.

**Fig. 2:**
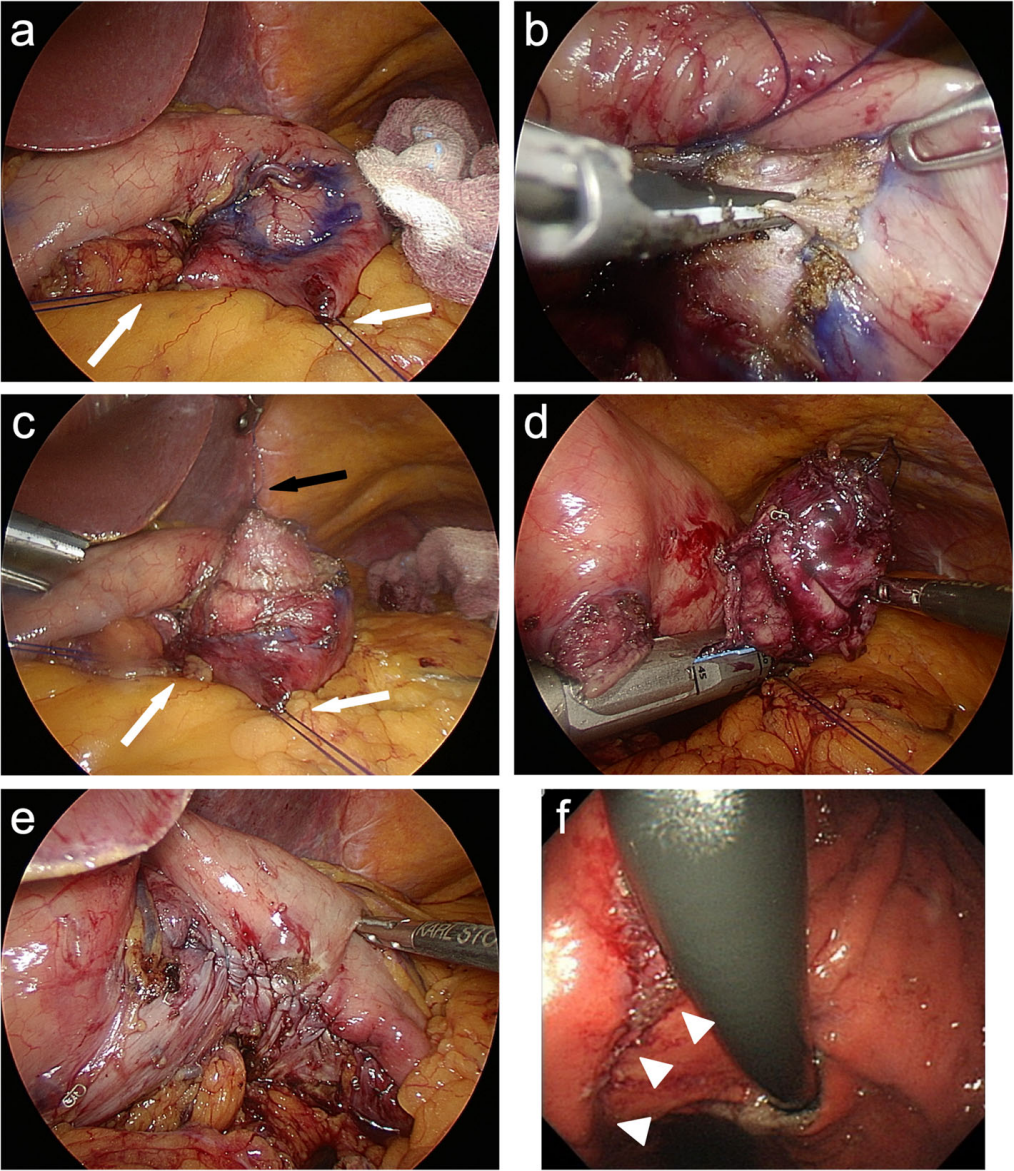
Operative findings. **a** After placing anchor sutures around the tumor to secure the surgical field (white arrows), we marked the margin line around the SMT from outside of stomach by pushing the margin with endoscopy. **b**, **c** We cut the seromuscular layer along the line using an ultrasonically activated device pulling an anchor suture to the tumor (black arrow). **d** We pulled the SMT out of the stomach and resected it by cut-and-closure procedure to mucosal layer using a laparoscopic stapling device three times. **e** We added seromuscular closure by handsewn suturing for reinforcement. **f** We confirmed the closure of the suture and the absence of bleeding with endoscopy during surgery (white arrow heads)

A camera port was inserted through an umbilical incision, then four additional ports (three 5-mm ports and one 12-mm port) were inserted into the upper abdomen in the shape of an inverted trapezoid under a pneumoperitoneum of 10 mmHg. The location of the SMT was not detected from the abdominal cavity. Therefore, endoscopy was used to identify the tumor location. The tumor was located at the posterior wall near the cardia of the stomach. We opened the omental bursa and used anchor sutures around the tumor to secure the surgical field. We then marked the margin line around the SMT from outside the stomach by pushing the margin with the endoscope (Fig. 2a). Next, we cut the seromuscular layer along the line using an ultrasonically activated device, pulling an anchor suture to the tumor (Fig. 2b, c). After that, we pulled the SMT out of the stomach and resected it by a cut-and-closure procedure to the mucosal layer using a laparoscopic stapling device three times (Fig. 2d). After adding seromuscular closure by handsewn suturing for reinforcement (Fig. 2e), we removed the SMT from the camera port using a single-use specimen pouch. We confirmed the closure of the suture and the absence of bleeding with endoscopy during surgery (Fig. 2f). The operating time was 198 min, and blood loss was minimal.

**Fig. 3:**
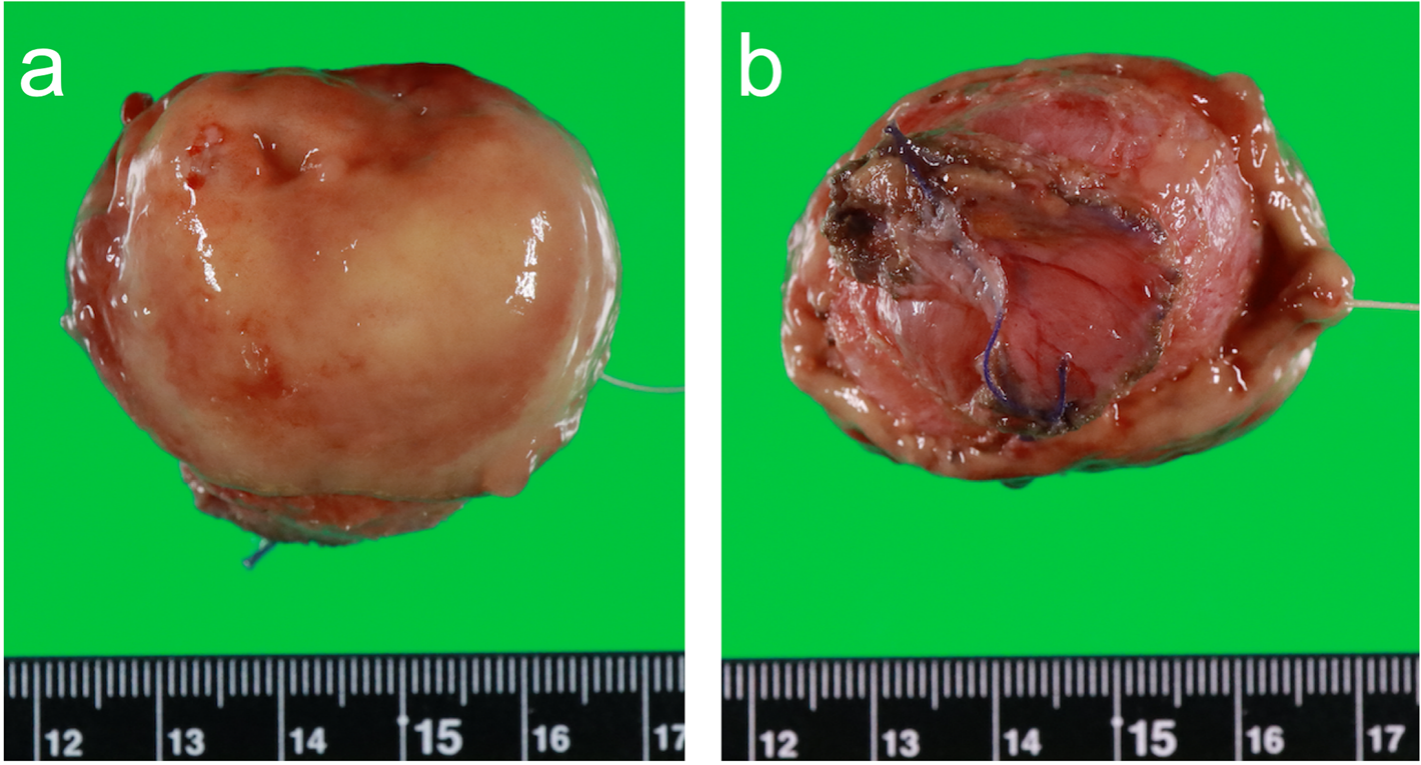
Operative specimen. The tumor was 37 × 31 mm. **a** Mucosal side. **b** Serosal side

**Fig. 4:**
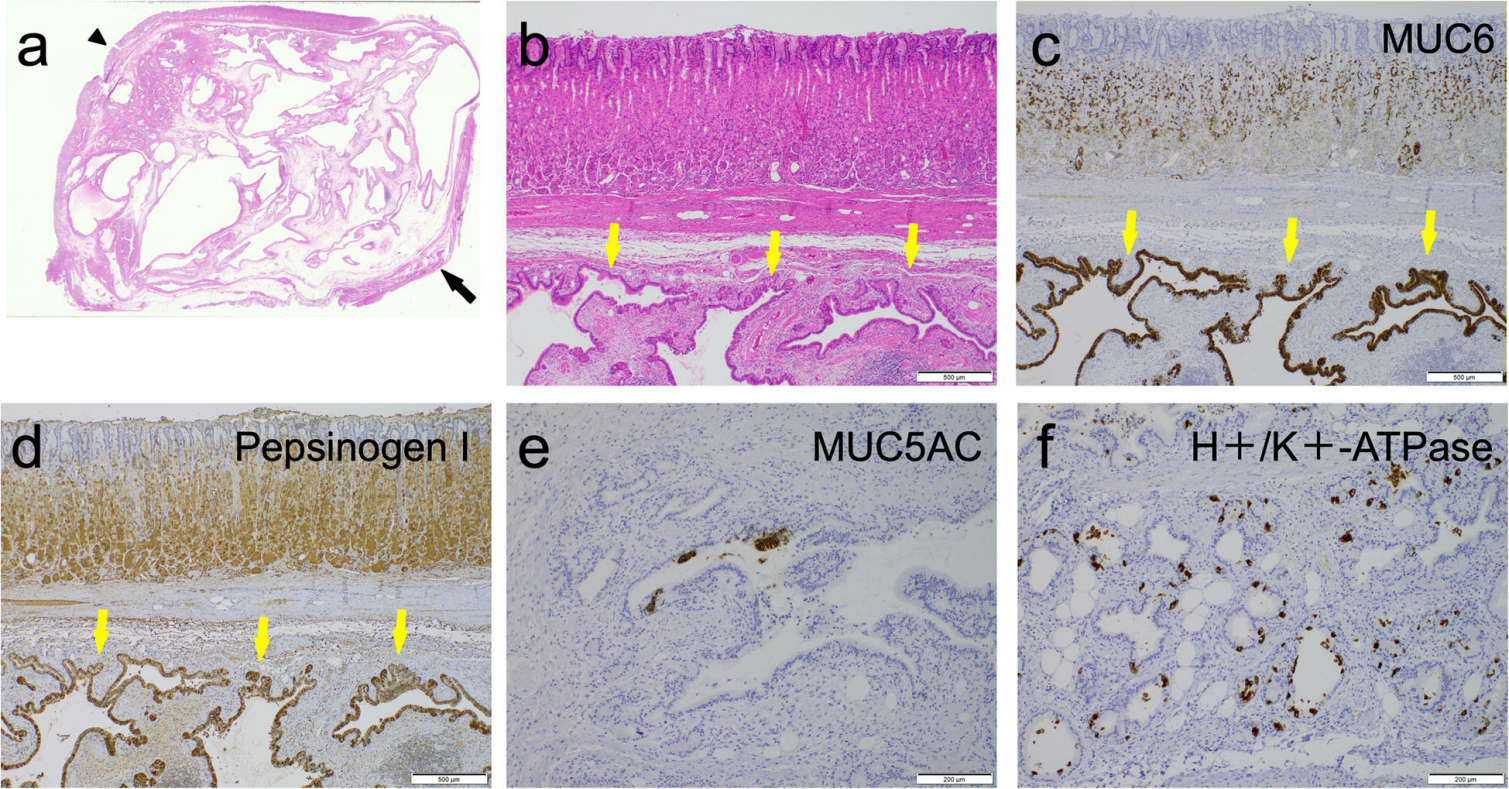
Histopathological findings. Epithelium (arrowhead). Cystic irregular dilated, large glands (yellow arrows). **a** Hematoxylin and eosin staining of the tumor (Loupe view). Proliferation of the enlarged glands with cystic dilatation and bands of smooth muscular fibers (black arrow) in the submucosa. **b** Hematoxylin and eosin staining of the tumor (× 40 magnification). **c** Immunohistochemical staining of MUC6 (× 40 magnification). **d** Immunohistochemical staining of Pepsinogen I (× 40 magnification). **e** Immunohistochemical staining of MUC5AC (× 100 magnification). **f** Immunohistochemical staining of H^+^/K^+^-ATPase (× 100 magnification)

The resected specimen was a cavernous tumor measuring 37 × 31 mm in the stomach wall (Fig. 3a, b). Histopathological examination showed the proliferation of enlarged glands with cystic dilatation and some bands of smooth muscle fibers such as the muscular layer of mucosa in the submucosa (Fig. 4a, b). There were no spindle cells associated with GIST. Immunohistological examination revealed positive expressions of MUC6, Pepsinogen I, MUC5AC, and H^+^/K^+^-ATPase, in glandular epithelium cells in the submucosa, which are the markers of mucous neck cells, chief cells, foveolar epithelium, and parietal cells, respectively (Fig. 4c–f). These features revealed that both the foveolar epithelium and the fundic gland were present in the submucosa, and suggested the diagnosis of GHIP. The patient had an uneventful postoperative course and was discharged on the ninth postoperative day.

## Discussion

Although several reports have addressed the various treatments of GHIP, such as ESD, gastrectomy, laparoscopic wedge resection, or LECS [4, 5, 9, 10], to the best of our knowledge, this is the first case of GHIP that was diagnosed and treated using modified CLEAN-NET.

GHIP is rare, making up for less than 1% of all gastric polyps [11, 12]. It is pathologically defined by the inverted growth of the hyperplastic gastric mucosal components into the submucosa and smooth muscle located in the submucosal layer, with branching from the proliferation of smooth muscle bundles [13, 14]. In the present case, we confirmed such findings by pathological examination, including immunohistochemical staining. GHIP is thought to occur due to the infiltration of the mucosa through muscularis mucosa cracks or defects caused by repeated erosion [15]. There are two types of hamartomatous inverted polyps: SMT type, as in the present case, which does not have a stalk; and polyp type, which has a stalk [9].

GHIP is usually asymptomatic and tends to be found incidentally, despite occasionally manifesting as an intestinal obstruction or as anemia secondary to chronic blood loss [9]. It sometimes develops into an SMT of more than 2 cm in diameter or an SMT with a central dimple. Ueo et al. reported that the observation of the multilocular anechoic region in the third layer of the gastric wall in endoscopic ultrasound (EUS) examination might be suggestive of GHIP [16]. This feature could distinguish GHIP from other SMT like GIST. However, other features of EUS imaging also have been reported, such as diffuse hyperechoic mass located in the submucosal layer [17]; thus, it is impossible to diagnose GHIP by EUS alone. In this case, a heterogenous tumor with cystic spots was shown by EUS but its location in gastric wall layers was unclear, while we were not familiar with its image of EUS. Thus, we could not diagnose the SMT as GHIP by EUS before resection. In contrast, endoscopic ultrasound-guided fine-needle aspiration (EUS-FNA) was not performed in this case. However, even though the tissue is taken correctly, making an accurate diagnosis is difficult because the foveolar epithelium of the GHIP can be misdiagnosed as gastric mucosal epithelium [18]. Previous studies also reported that it was difficult to diagnose before resection because of its inverted growth into the submucosal layer, as well as the paucity of previous case reports [4, 14].

GHIP is a benign tumor; however, there have been reports on GHIP that contained gastric cancer. Although its occurrence is very low, and the association of GHIP with carcinogenesis is controversial, it should be considered [5, 6]. In addition, it is reported that GHIP sometimes has a central dimple as admitted on GIST [4, 7]. In our case, a central dimple was also admitted. We suspected the tumor as GIST. Thus, an en bloc resection was needed for both the diagnosis and treatment of the tumor. Therefore, in the present case, it was important not only to resect the tumor completely and perform a less invasive diagnosis and treatment, but also to prevent tumor dissemination. We therefore conducted modified CLEAN-NET.

Original CLEAN-NET, which was first reported by Inoue et al. in 2012, is one of the modified LECS procedures. The technique preserves the mucosal layer, which provides a mechanical barrier between the gastric lumen and the peritoneal cavity. Therefore, it can avoid intraoperative tumor dissemination and exposure to the content of the stomach [8, 19]. Non-exposed endoscopic wall-inversion surgery (NEWS) is also a non-exposure technique among modified LECS procedures [20]. However, since the tumor is retrieved orally in NEWS, as with ESD, it can only be used for tumors sized < 3 cm. On the other hand, original CLEAN-NET can be performed for tumors sized ≥ 3 cm, because the tumor is removed transabdominally; however, the risk of deformation of the remnant stomach is high when the tumor size is large [20].

In contrast, there are limitations to CLEAN-NET. Resection of a tumor located adjacent to the esophagogastric junction (EGJ), the pyloric ring, or the lesser curvature is sometimes technically demanding, because the likelihood of the occurrence of postoperative stenosis caused by deformity of the stomach is higher in these locations. In addition, a tumor located on the posterior wall was also reported to be difficult to remove by CLEAN-NET, due to its inaccessibility [20]. Fujishima et al. reported a modified CLEAN-NET technique, modifying the resection and closure procedure using laparoscopic stapling devices from full-layer stapling to only mucosal layer and adding seromuscular closure by handsewn suturing. This technique enabled resect the gastric SMTs near the EGJ or the pyloric ring without any stenosis [21]. In our case, the tumor was over 3 cm and located at the posterior wall near the cardia of the stomach. Thus, we selected modified CLEAN-NET and could remove the tumor with little deformation of the stomach relatively easily by using anchor sutures around the tumor to secure the surgical field.

Modified CLEAN-NET is one of the potential treatment modalities for GHIP, in terms of conducting en bloc resection because it avoids dissemination, is minimally invasive, and has a reduced chance in resulting in deformity of the stomach.

## Conclusion

In this case, we found modified CLEAN-NET to be a feasible procedure with a non-exposure technique for GHIP with a central dimple that was not able to be removed orally. The clinical application of modified CLEAN-NET will be expanding to various types of tumors by taking account of its advantages and limitations.

## Data Availability

Data sharing is not applicable to this article, as no datasets were generated or analyzed during the current study.

## Abbreviations

GHIP: Gastric hamartomatous inverted polyp
CLEAN-NET: Combination of laparoscopic and endoscopic approaches to neoplasia with non-exposure technique
SMT: Submucosal tumor
ESD: Endoscopic submucosal dissection
LECS: Laparoscopic and endoscopic cooperative surgery
EUS: Endoscopic ultrasound
EUS-FNA: Endoscopic ultrasound-guided fine-needle aspiration
NEWS: Non-exposed endoscopic wall-inversion surgery
EGJ: Esophagogastric junction
